# Spirituelle Interventionen in der multimodalen Schmerztherapie

**DOI:** 10.1007/s00482-024-00788-z

**Published:** 2024-01-15

**Authors:** Kristin Kieselbach, Ursula Frede

**Affiliations:** 1 Interdisziplinäres Schmerzzentrum ISZ, Universitätsklinikum Freiburg, Breisacher Str. 117, 79106 Freiburg, Deutschland; 2 Hofgasse 2a, 78337 Öhningen, Deutschland

**Keywords:** Chronischer Schmerz, Spiritualität, Wertorientierung, Sinn des Lebens, Biopsychosozial-spirituelles Modell, Chronic pain, Spirituality, Value orientation, Meaning of life, Biopsychosocial-spiritual model

## Abstract

Verstehen wir chronischen Schmerz nicht nur als Krankheit, sondern zugleich als existenzielle Krise, erscheint eine Berücksichtigung spiritueller Aspekte im Behandlungsprozess als ebenso folgerichtig wie sinnvoll. Spiritualität wird als Oberbegriff für alle Aktivitäten und Erfahrungen verstanden, die dem Leben des Menschen Sinn und Bedeutung verleihen – unabhängig von seiner religiösen Zugehörigkeit. Bislang wurden spirituelle Aspekte therapeutisch hauptsächlich im palliativen Kontext berücksichtigt. Aktuellen umfragebasierten Erhebungen bei Schmerzerkrankten zufolge führt die Einbeziehung der spirituellen Thematik in die Therapie zu einer Verbesserung von Lebensqualität und Schmerztoleranz und wird überdies von den Betroffenen explizit gewünscht. Eine konsequente Erweiterung multimodaler Behandlungsansätze im Sinne eines biopsychosozial-spirituellen Konzepts wurde bislang noch nicht umgesetzt. Relevant für die praktische Umsetzung sind v. a. folgende Grundhaltungen und Verhaltensweisen: Offenheit für spirituelle Themen und Authentizität, Erhebung der spirituellen Anamnese, Zuhören, Standhalten, Aktivierung von Werten, Rückgriff auf Motive aus Religion, Mythologie und Kunst. Die fachliche Zuständigkeit betrifft generell alle Behandler, kann aber auch qualifizierte Fachpersonen für einen spezialisierten Beistand erfordern. Die Integration einer authentischen spirituellen Begleitung in die multimodale Schmerztherapie soll über eine Ressourcenaktivierung und die Identifikation belastender spiritueller Überzeugungen dazu beitragen, Selbstwert- und Identitätserleben der Betroffenen zu stabilisieren. Die detaillierte Integration und die Untersuchung der Effizienz spiritueller Interventionen in der multimodalen Schmerztherapie bedürfen weiterer Untersuchungen.

## Abstract (en)

If we understand chronic pain not only as a disease but also as an existential crisis, it seems logical and reasonable to consider spiritual aspects in the treatment process. Spirituality is understood as an umbrella term for all activities and experiences that give meaning and significance to people’s lives—irrespective of their religious affiliation. So far, spiritual aspects have been considered therapeutically mainly in the palliative context. According to current survey-based studies of pain patients, the inclusion of spiritual themes in therapy leads to an improvement in quality of life and pain tolerance and is moreover explicitly desired by those patients. A consistent expansion of multimodal treatment approaches in the sense of a biopsychosocial-spiritual concept has not yet been implemented. The following basic attitudes and behaviors are relevant for practical implementation: openness to spiritual themes and authenticity, taking a spiritual history, listening, standing firm, activation of values, use of motives from religion, mythology, and art. Professional competence generally involves all practitioners, but may also require qualified professionals for specialized assistance. The integration of authentic spiritual assistance into multimodal pain management should help to stabilize self-esteem and the experience of identity of the patients through resource activation and identification of burdensome spiritual beliefs. The detailed integration and investigation of the efficiency of spiritual interventions in multimodal pain therapy require further research.

## Einführung

Die interdisziplinäre multimodale Schmerztherapie gehört seit vielen Jahren zum Standardrepertoire zahlreicher schmerztherapeutischer Einrichtungen in Deutschland [28]. Zentral für den multimodalen Behandlungsansatz ist das teamintegrierte Zusammenwirken schmerzspezifischer medizinischer, psychotherapeutischer und physiotherapeutischer Verfahren auf der Basis eines biopsychosozialen Verständnisses chronischer Schmerzen. Alle beteiligten Professionen orientieren sich am übergeordneten Ziel einer „Verbesserung der objektiven und subjektiven Funktionsfähigkeit“ der Betroffenen [4].

Dieses Ziel allein muss hinterfragt werden, weil es Menschen gibt, deren Funktionstüchtigkeit nur bedingt oder gar nicht mehr verbessert werden kann, die aber noch viele Jahre mit Schmerzen werden leben müssen. Auch Betroffene, bei denen eine gewisse Verbesserung der Funktionsfähigkeit erreicht werden kann, leiden unter einer Erfahrung, die mit folgendem Satz zusammengefasst werden kann: „Mein Leben ist aus den Fugen geraten.“ Das aber heißt: Chronischer Schmerz ist nicht nur ein *gesundheitliches Problem*, sondern zugleich immer auch eine *existenzielle Krise* [27]. Eine Sicht auf chronischen Schmerz als Störungsbild mit somatischen und psychischen Faktoren (ICD-10-GM-Code F45.41) hat überwiegend informierende, aktivierende und übende Maßnahmen zur Folge. Chronischer Schmerz, der als Krankheit *und* als existenzielles Widerfahrnis betrachtet wird, bedarf über den Einsatz dieser Maßnahmen hinaus auch einer Einbeziehung der *Spiritualität*.

Anliegen der vorliegenden Arbeit ist es, dem Thema Spiritualität in der multimodalen Therapie chronischer Schmerzen den Nimbus des Außergewöhnlichen zu nehmen. Nach einer kurzen Übersicht zum Stand der Forschung sowie zum Verständnis von Spiritualität folgt eine Auseinandersetzung zu Fragen der Zuständigkeit und den Zielen bei einer Integration spiritueller Aspekte in den Behandlungsprozess. Anschließend werden Grundhaltungen und Verhaltensweisen diskutiert, die für eine solche Integration wesentlich sind. Überlegungen zur erweiterten Rolle der Behandelnden beenden den Artikel.

## Zum Stand der Forschung

Religiöse ebenso wie spirituelle Themen wurden in Medizin und Psychotherapie lange Zeit tabuisiert [54]. Seit ca. 1990 jedoch häufen sich Untersuchungen, wonach sich die Berücksichtigung von Spiritualität positiv auf die körperliche und emotionale Gesundheit von Menschen auswirken kann [5]. Im Bereich der Medizin findet die spirituelle Thematik bislang hauptsächlich im Rahmen palliativer Versorgung sterbender Menschen Beachtung [23]. In Psychiatrie und Psychotherapie steigt die Zahl der Veröffentlichungen zur Bedeutung von Spiritualität stetig an [13], doch wird die Frage ihrer Integration nach wie vor kontrovers diskutiert.

In theologisch-religionswissenschaftlicher und seelsorgerischer Hinsicht kann im deutschen Sprachraum bereits auf relevante Publikationen Bezug genommen werden, die sich hauptsächlich mit Schmerz und Spiritualität am Lebensende und im palliativmedizinischen Kontext befassen [47, 56]. Bezogen auf die Schmerztherapie stellt sich die gegenwärtige Situation folgendermaßen dar: Viele Studien, überwiegend aus dem angloamerikanischen Raum, haben gezeigt, dass spirituelle Aktivitäten das allgemeine Wohlbefinden schmerzkranker Menschen steigern sowie zu verbesserter Lebenszufriedenheit und erhöhter Schmerztoleranz führen [32, 50]. Davon ausgehend plädieren einige Autoren für einen *biopsychosozial-spirituellen* Ansatz zur Behandlung chronischer Schmerzen [16]. Diese Forderung entspricht dem Ergebnis jüngster Befragungen von Schmerzpatienten, wonach die meisten Betroffenen eine Einbeziehung spiritueller Aspekte in ihre Behandlung explizit wünschen [6, 20, 46]. Zugleich weisen andere Studien darauf hin, dass bislang nur wenige Kliniker auf die spirituellen Fragen und Nöte ihrer Patienten eingehen [3, 45].

## Begriffliche Klärung

Im alltäglichen Sprachgebrauch werden Begriffe rund um Spiritualität häufig in spiritistische, metaphysische und esoterische Zusammenhänge gestellt oder mit Religion gleichgesetzt. In der Fachliteratur weist allein schon die Vielzahl unterschiedlicher Definitionen auf die Komplexität dieses Begriffs hin (z. B. [43, 44, 50, 54]). Der Palliativmediziner Borasio bringt diese Komplexität auf den Punkt: „Jeder Versuch, Spiritualität zu definieren, ist zum Scheitern verurteilt“ [7]. Einige Autoren betrachten Spiritualität als Synonym für Religiosität, manche bemühen sich um eine deutliche Unterscheidung beider Begriffe, wieder andere verstehen Spiritualität als den umfassenderen Begriff, der „sich sowohl auf eine überweltliche (göttliche) Dimension als auch auf nichtmaterielle Dimensionen (wie Humanität, Familie, Freundschaft, Natur, Kunst, Arbeit, Selbstentfaltung) […] beziehen kann“ [23]. Im Rahmen palliativer Versorgung wird Spiritualität überwiegend „als Verbundenheit einer Person mit dem [verstanden], was ihr Leben trägt, inspiriert und integriert, sowie die damit verbundenen existenziellen Überzeugungen, Werthaltungen, Erfahrungen und Praktiken, die religiöser oder nicht-religiöser Art sein können“ [38]. Bezogen auf das Gesundheitswesen im Allgemeinen haben sich internationale Experten im Rahmen der Konsensuskonferenz „On Improving the Spiritual Dimension of Whole Person Care“ auf folgende Definition von Spiritualität geeinigt: „Spirituality is a dynamic and intrinsic aspect of humanity through which persons seek ultimate meaning, purpose, and transcendence, and experience relationship to self, family, others, community, society, nature, and the significant or sacred. Spirituality is expressed through beliefs, values, traditions, and practices“ [44]. Zur ausführlichen Auseinandersetzung mit dem vielschichtigen Begriff der Spiritualität verweisen wir beispielsweise auf [1, 56].

Vorherrschend für das Konzept der Spiritualität und *gemeinsames Merkmal* fast aller Definitionen sind Begriffe, die für die *Identität* des Menschen bedeutsam sind, insbesondere für sein Bemühen um *Sinngebung* und *Wertsetzung* [50]. Von diesen Kernbegriffen ausgehend wird Spiritualität im vorliegenden Text als Bemühen darum verstanden, dem eigenen Leben Sinn und Bedeutung zu geben, sich bei der Gestaltung des Lebens an persönlichen Werten und Vorstellungen davon zu orientieren, was wirklich wichtig ist. Dieses Verständnis von Spiritualität entspricht in etwa der Definition durch die *Spiritual Care Task Force der European Association of Palliative Care *(EAPC): „Spirituality is the dynamic dimension of human life that relates to the way persons (individual and community) experience, express and/or seek meaning, purpose and transcendence, and the way they connect to the moment, to self, to others, to nature, to the significant and/or the sacred“ [35]. So definiert ist Spiritualität „als eine anthropologische Kategorie“ zu sehen, das heißt als ein Aspekt, der zum Menschsein gehört [54]. Eine Schmerztherapie, die den Menschen in seiner Gesamtheit erfassen will, sollte sich deshalb nicht auf biologische, psychologische und soziale Faktoren beschränken, vielmehr auch seine spirituellen Komponenten einbeziehen, wie v. a. seine Wert- und Sinnvorstellungen. Anders formuliert: Wenn chronischer Schmerz zu Sinn- und Identitätskrisen führt [27] und Spiritualität entscheidend zur Bewältigung von Krisen dieser Art beitragen kann [13], sollte diese integraler Bestandteil einer Behandlung schmerzkranker Menschen sein.

## Wer ist zuständig für die Einbindung von Spiritualität in die Schmerztherapie?

Was Borasio [7] für die palliative Versorgung formuliert, gilt gleichermaßen für die Versorgung von Schmerzpatienten, deren Sterben nicht kurz bevorsteht: „Spiritualität ist Teamarbeit.“ Einer aktuellen Patientenbefragung zufolge können sich schmerzkranke Menschen „alle Fachpersonen aus allen Fachrichtungen als Ansprechpartner für spirituelle Themen im Rahmen ihres Behandlungsprozesses vorstellen“ [46]. Beispielsweise werden benannt: *„Das Pflegepersonal auf der Station. Das hat am meisten Kontakt“* […]. *„Also eine Physiotherapeutin […] Dort kann ich nackt vor ihr stehen. Was ich bei der Ärztin nicht unbedingt gern mache“ *[46]. Zugleich ist den Betroffenen bewusst, dass nicht alle Fachpersonen über die notwendigen Fähigkeiten und/oder Möglichkeiten verfügen, sich mit spirituellen Fragen und Nöten tiefer gehend zu befassen.

Im Rahmen einer Befragung von Fachpersonen zur Integration spiritueller Aspekte in die multimodale Schmerztherapie unterscheiden die Teilnehmenden „zwischen einem generellen und einem spezialisierten Beistehen“ [45]. Auch in den neuen „Leitlinien zu Spiritual Care in Palliative Care“ [38] wird betont, dass es im Umgang mit der spirituellen Dimension von Patienten sowohl gemeinsame als auch professionsspezifische Aufgaben gibt, wobei hier zwischen „seelsorglicher und gesundheitsberuflicher Spiritual Care“ unterschieden wird [36].

Eine Differenzierung zwischen generellem und spezialisiertem Beistehen würde sowohl den Bedürfnissen der Patienten als auch den Möglichkeiten der Behandelnden Rechnung tragen. Alle Fachpersonen sollten *generellen Beistand* leisten können, d. h. dazu bereit sein, sich auch auf spirituelle Anliegen eines Patienten einzulassen. Zugleich sollten sie die Grenze eigener Kompetenz in diesem Bereich reflektieren und einen Spezialisten für spirituelle Betreuung hinzuziehen, sollte diese Grenze erreicht sein. Für einen *spezialisierten Beistand* sind von der Qualifikation her Fachpersonen aus den Bereichen *Krankenhausseelsorge, Psychotherapie, Medizin und Pflege* geeignet. Die Indikationsstellung für die Hinzuziehung speziell seelsorgerischer Kompetenz könnte auch durch ein kürzlich entwickeltes Instrumentarium unterstützt werden [48].

Inzwischen gibt es ein breites Angebot an berufsbegleitender Weiterbildung in Block‑, Wochenend- und Online-Kursen. Für die Fort- und Weiterbildung in spiritueller Begleitung von Palliativpatienten hat die Deutsche Gesellschaft für Palliativmedizin bereits zentrale Lernziele und Unterrichtsthemen formuliert. Verwiesen sei in diesem Zusammenhang auf Gratz und Roser [17]. Bisherige Effektivitätsstudien zeigen, dass sich bereits kurze Schulungen in spiritueller Betreuung positiv auf die spirituelle Kompetenz und das Wohlbefinden von Fachpersonen auswirken können [25]. Für die Integration von Spiritualität in die Begleitung von Menschen, deren Lebenserwartung nicht begrenzt ist, gibt es bislang (noch) keine Ausbildungsangebote. Zunächst gilt es, das Bewusstsein für die Notwendigkeit einer solchen Einbeziehung zu fördern. Davon unbenommen sollten für beide Arten des Beistands zur Vermeidung kognitiver und emotionaler Überforderung der Behandelnden begleitende Supervisionen sowie gezielte Trainingseinheiten angeboten werden, zu Themen wie: Reflexion der Bedeutung, die spirituelle Themen für schmerzkranke Menschen haben können, Klärung eigener spiritueller Werte und Überzeugungen, Vermittlung und Vertiefung von Grundkenntnissen in spirituellen und religiösen Weltanschauungen sowie Förderung von Kompetenzen, wie sie in den folgenden Kapiteln beschrieben werden.

Abschließend sei betont: Die Einbindung von Spiritualität in die Schmerztherapie stellt keine Aufgabe dar, die unnötige Kapazitäten bindet. Eher im Gegenteil. Kann doch eine solche Einbindung entscheidend dazu beitragen, „Patient/-innen in ihrer Schmerzsituation besser verstehen und sie in einem hilfreichen Umgang mit ihrer chronischen Schmerzerkrankung begleiten zu können“ [45].

## Ziele einer Integration von Spiritualität in die Schmerztherapie

Eine Einbindung von Spiritualität in die multimodale Schmerztherapie erscheint v. a. mit Blick auf folgende Ziele wichtig:

- Durch Integration der spirituellen Perspektive können Ressourcen aktiviert werden, die den Betroffenen helfen, ihrem Leben Sinn und Bedeutung zu geben, Vertrauen in die Kontinuität ihrer Identität zu entwickeln – trotz vielfältiger Veränderungen ihrer Lage.
- Eine möglichst frühzeitige Identifikation belastender spiritueller Überzeugungen (z. B. Schmerz als Strafe) trägt dazu bei, bestimmte Reaktionen von Patienten besser verstehen und sie bei der Entwicklung entlastender Sichtweisen unterstützen zu können.
- Ein Erkennen sogenannter „spiritueller Schmerzen“ ist allein schon deshalb von Bedeutung, weil gegenüber diesem Schmerz jedes Medikament wirkungslos bleibt und Ziele wie die Verbesserung der Funktionsfähigkeit nicht greifen. Spiritueller Schmerz, definiert als Ausdrucksphänomen, „in dem sich der Zusammenbruch orientierungsstiftender Lebens- und Glaubensgewissheit“ zeigt [37], kann zu körperlichen Schmerzen führen oder bestehende Schmerzen intensivieren. Bleiben die Hintergründe dieser Schmerzen unerkannt, ist zu befürchten, dass die Interventionen multimodaler Schmerztherapie großenteils ohne Wirkung sein werden.

### *Zusammengefasst*.

Unabhängig von der Individualität therapeutischer Zielsetzungen (in Abhängigkeit von der Person und Situation des Patienten) wird das übergeordnete Anliegen einer Berücksichtigung von Spiritualität darin gesehen, Quellen der Sinngebung zu aktivieren, sodass Erkrankte (wieder) in sich selbst Halt finden, ein Gefühl für den Wert ihrer Person entwickeln und bewahren können – *mit* chronischem Schmerz und *trotz* eingeschränkter Funktionstüchtigkeit.

## Einstellungen und Verhaltensweisen im Umgang mit spirituellen Anliegen schmerzkranker Menschen

In Deutschland werden Fachpersonen in der Schmerztherapie bislang kaum oder gar nicht auf das Thema Spiritualität vorbereitet. Hoffnung auf Änderung der Situation macht ein Positionspapier von Alt-Epping und Kollegen anlässlich der Reform der ärztlichen Approbationsordnung und Umsetzung des Nationalen Kompetenzbasierten Lernzielkatalogs Medizin [2]. In diesem Papier wird die Dringlichkeit einer Einführung von „Religiosität und Spiritualität“ in das Medizinstudium betont und ein Katalog von neun möglichen Lernzielen für die Lehre formuliert.

Seit Januar 2023 steht ein von der Professur für Spiritual Care der Universität Zürich entwickelter „Leitfaden zur Einbeziehung der spirituellen Dimension in die multimodale Schmerztherapie“ zur Verfügung [21]. Dieser Leitfaden liefert Hinweise zum Umgang mit dem Thema *Spiritualität in der Schmerztherapie*. Er konzentriert sich hauptsächlich auf Fragen zu bestimmten Bereichen, wie z. B. Krankheitskonzepten, spirituellen Ressourcen und spirituellen Belastungen. Die Fragen dienen zum einen dem „Gesprächseinstieg und der Exploration von spirituellen Ressourcen und Belastungen“, „einer gezielten Integration spiritueller Aspekte in die Behandlung“ zum anderen (ebd.). Alle Dokumente zum Leitfaden sind auf der Website www.spiritualcare-leitfaden.ch abrufbar. Haltungen in der Begegnung mit schmerzkranken Menschen wie „Offenheit, respektvolles Zuhören und Verstehen“ werden benannt, jedoch nicht konkret beschrieben.

Der Schwerpunkt der folgenden Kapitel liegt nun eben darauf: Einstellungen und Verhaltensweisen zu diskutieren, die uns für einen *generellen Beistand* bedeutsam erscheinen. Die beschriebenen Interventionen sind keineswegs neu. Doch sind sie *essenziell* dafür, dass schmerzkranke Menschen ihre spirituellen Anliegen ansprechen können. Grundlage unserer Überlegungen sind die Selbstaussagen Betroffener in Interviews [26, 40], Befragungsstudien [6, 46] und Autobiografien [30, 41].

### Therapeutische Offenheit für spirituelle Themen

Unabhängig von konkreten Verhaltensweisen zur Auseinandersetzung mit spirituellen Fragen schmerzkranker Menschen – von zentraler Bedeutung ist die *innere Haltung* der Behandelnden, gekennzeichnet v. a. durch folgende Aspekte [13, 45]:

- Reflexion eigener spiritueller Überzeugungen, persönlicher Sinn- und Wertsetzungen, z. B.: Worin sehe ich den Sinn meines Lebens? Welche Werte sind für mich wichtig? Woran glaube/worauf hoffe ich?
- Offenheit für die spirituellen Anliegen schmerzkranker Menschen verbunden mit der Bereitschaft, sich darauf einzulassen
- Vertrauen in die Ressourcen des Kranken, wie „verschüttet“ sie auch sein mögen
- Toleranz und Respekt gegenüber Sichtweisen, die anders sind als die eigenen
- Sensibilität für den Moment, an dem die Grenzen eigener Kompetenz in spirituellen Dingen erreicht sind

Die Aufgabe der Behandelnden besteht *nicht* darin, auf jede Frage eine Antwort zu haben. Vielmehr geht es darum, den Erkrankten bei der Suche nach eigenen Antworten zu begleiten (s. Abschn. „Zuhören“). Auch für die Behandelnden selbst kann Spiritualität eine wichtige Ressource sein, „um die Belastung durch die Konfrontation mit dem Leid der Patientinnen und Patienten besser bewältigen zu können“ [13]. Offenheit für spirituelle Themen lässt sich fördern – beispielsweise durch den Austausch mit Kollegen etwa im Rahmen einer Supervision, durch Interesse an den Selbstaussagen der Patienten, aber auch durch die Lektüre von Büchern, die Betroffene über den Umgang mit ihrer Erkrankung geschrieben haben (z. B. [41]). Entscheidend ist der Gedanke: „Ich möchte mehr über diese Dinge erfahren.“ Es mag banal klingen: Größter Feind für die Haltung der Offenheit ist die Routine. Hilfreich dagegen ist das Bemühen darum, jeden Patienten neu als individuelle Person in einer höchst spezifischen Situation kennenzulernen.

### Authentizität

In der bereits erwähnten Studie zur Einbindung von Spiritualität in die Schmerztherapie wiesen die befragten Schmerzpatienten explizit auf die „herausragende Rolle“ der Authentizität im Umgang mit spirituellen Themen hin [46]. Patienten sind meist sehr feinfühlig für *nichtsprachliche *Verhaltensweisen der Menschen in ihrer Umgebung, nehmen kleinste Veränderungen im Ausdruck ihres Gesprächspartners wahr, auch Zeichen, derer sich der andere unter Umständen gar nicht bewusst ist. Insbesondere auch die Einstellungen, die eine Fachperson ihrem Patienten gegenüber hat, werden größtenteils *nonverbal* vermittelt – über Stimme, Sprechweise, Mimik und Gestik, wobei die Überzeugungskraft ihrer *nonverbalen* Hinweise deutlich mehr wiegt als die ihrer Worte. Man kann nicht authentisch *tun*. Authentisch kann man nur *sein*, indem man sich bemüht, nach eigenen Werten zu handeln, offen und ehrlich zu sich selbst und anderen zu sein [28].

Wie aber lernt man Authentizität? Oder hat man sie? Beides: Man hat Authentizität, kann sie aber auch in entsprechenden Kursen, Workshops und Seminaren fördern. Zentrale Voraussetzung authentischen Verhaltens ist, das eigene Wertesystem zu kennen, vertraut zu sein mit dem, was einem wichtig ist.

Auf die Authentizität des Behandelnden kommt es v. a. in zwei Situationen an:

- Zum einen in Momenten großer *emotionaler Dichte*, in denen das Erleben des Patienten den Behandelnden gefühlsmäßig stark „berührt“. In einer solchen Situation kann sich Authentizität – je nach Persönlichkeit – beispielsweise im Ausdruck mitfühlender Anteilnahme zeigen oder auch in betroffenem Schweigen.
- Zum anderen ist die Aufrichtigkeit der Fachperson dann gefragt, wenn sie sich angesichts der spirituellen Anliegen eines schmerzkranken Menschen überfordert fühlt. Die meisten Patienten haben Verständnis, wenn sie aus diesem Grund an ein anderes Mitglied des interprofessionellen Teams überwiesen werden: *„Ich fände es keine Schande, wenn ein Arzt mal sagt, mein Fachwissen hört da auf“* [46].

Authentizität angesichts chronischer Schmerzen und das Aufrechterhalten von Hoffnung schließen einander nicht aus! Der Patient hofft auf Schmerzlinderung, vor allem aber darauf, ein Mensch von Wert zu sein und zu bleiben, als solcher anerkannt und behandelt zu werden. In *dieser* Hoffnung kann ein Patient auch dann bestärkt werden, wenn sich am Verlauf seiner Schmerzen womöglich kaum etwas ändern lässt: Ein Arzt oder Therapeut, der die Grenzen medizinisch-psychologischer Möglichkeiten ebenso ehrlich anzuerkennen vermag wie seine persönlichen Grenzen, *solidarisiert* sich mit dem Patienten, beispielsweise indem er sagt: „Ich wünschte, es wäre anders. Doch ich weiß nicht, wie es mit Ihren Schmerzen weitergehen wird. Was ich jedoch weiß ist, dass ich auch weiterhin mit Ihnen nach einem Weg suchen werde, wie Sie damit leben können.“ Worte dieser Art erleichtern es dem Patienten, sich mit seiner augenblicklichen Realität auseinanderzusetzen – statt mit Trugbildern illusorischer Hoffnung oder der belastenden Vorstellung, persönlich versagt zu haben. Zugleich vermitteln sie ihm die Erfahrung, ernst genommen und respektiert zu werden.

### Einbeziehung der spirituellen Anamnese

Eine spirituelle Anamnese hat vorrangig zum Ziel, „Bedürfnisse, Probleme, aber auch Ressourcen zu erkennen, die sich aus der Spiritualität/Religiosität des Patienten ergeben, vor allem in Hinblick auf Krankheitsverarbeitung und Lebensqualität“ [12]. Lassen sich in den Aussagen eines Patienten weder direkte noch indirekte Hinweise auf seine Glaubens- und Wertüberzeugungen finden, sollte man gezielt danach fragen, allein schon, um ihm zu vermitteln: „Hier darf über alles geredet werden, auch über Sinnfragen und Glaubensdinge.“ Fragen folgender Art bieten sich an [6]:

- Was ist Ihnen wirklich wichtig im Leben?
- Woran hängt Ihr Herz?
- Wer oder was gibt Ihnen Halt im Leben?

Da Menschen eine höchst unterschiedliche Auffassung von Spiritualität haben, sind konkrete Fragen dieser Art einer direkten Frage nach der persönlichen Spiritualität vorzuziehen [40].

Eine Anamnese über spirituelle Orientierungen ist in zweierlei Hinsicht wichtig: Zum einen kann sie dazu beitragen, Ressourcen des Kranken zu aktivieren. Zum anderen können belastende spirituelle Überzeugungen erkannt und in die Behandlung einbezogen werden. Beispielsweise gibt es Menschen, die ihren Schmerz als Bewährungsprobe oder als Sühne für früheres Fehlverhalten erleben [45]. Andere Patienten sind davon überzeugt, dass bestimmte therapeutische Maßnahmen unvereinbar mit ihrem Glauben seien. Vorstellungen dieser Art können zur zusätzlichen Herausforderung werden, den Einsatz therapeutischer Maßnahmen erschweren oder sogar verhindern [11]. Nicht um eine Korrektur von Glaubensüberzeugungen geht es, vielmehr darum, sie zu respektieren und ernst zu nehmen, auch dann, wenn man diese nicht teilt. Wertschätzung und Akzeptanz einer Fachperson vermitteln dem Patienten Erfahrungen der Sicherheit und Entspannung, die seiner Angst entgegenwirken und sein körperlich-seelisches Wohlbefinden verbessern können [18].

Zur Erhebung der spirituellen Ressourcen und Belastungen eines Menschen sind eine Vielzahl unterschiedlicher Verfahren entwickelt worden:

- Interviewleitfäden (Assessment-Instrumente), wie beispielsweise das „Neumünster Assessment für Spiritual Care im Alter“ (NASCA; [42]), das halbstrukturierte, klinische Interview „SPIR“ [22] sowie der bereits erwähnte „Leitfaden zur Einbeziehung der spirituellen Dimension in die multimodale Schmerztherapie“ [21]
- Fragebögen wie beispielsweise „The Spiritual Needs Questionnaire“ [9] oder „The spiritual distress and resources questionnaire“ [39]

Jedoch sei betont: Spirituelle Erfahrungen und Gedanken sind zutiefst intim und privat. Darüber zu reden, setzt Vertrauen in das Gegenüber voraus. Fragebögen oder Interviewleitfäden können also bestenfalls begleitend, jedoch nicht als einziger Weg eingesetzt werden, um etwas über die Spiritualität eines Patienten zu erfahren.

### Zuhören

In einer Studie zur Integration von Spiritualität in die Schmerzbehandlung äußerten die meisten Betroffenen den Wunsch, „im Behandlungsprozess einen von Wertschätzung geprägten Raum zu haben, in dem auch spirituelle Themen zur Sprache kommen dürfen“ [46]. Ein solcher Raum entsteht für einen Patienten v. a. dadurch, dass man ihm *zuhört*. Zuhören ist mehr als „nur“ eine auditive Wahrnehmung, ist immer auch eine Leistung der Aufmerksamkeit, Beobachtung und Empathie [19]. Vereinfacht lassen sich zwei Formen des Zuhörens voneinander unterscheiden: zum einen das *selektive Zuhören*, bei dem der Zuhörende v. a. die Botschaften hört, die er für hörenswert hält, die seinen eigenen Erwartungen und Deutungsmodellen entsprechen [24]; zum anderen das *therapeutische Zuhören*, bei dem der Zuhörende auf das hört, „was der Patient von sich aus berichtet“ [24]. Kennzeichnend für diese Art des Zuhören sind v. a. folgende *Merkmale *[14]:

- Voraussetzung für wertschätzendes Zuhören ist eine *Begegnung mit dem Patienten auf Augenhöhe* – eine Begegnung, die durch das Bemühen gekennzeichnet ist, ihn (den Betroffenen) und seine Sicht der Dinge verstehen zu *wollen.*
- Zuhören bezieht sich nicht nur auf die Worte des Kranken, sondern ebenso auf seine *Mimik und Gestik*, auf Pausen im Gespräch, ein Blasser- oder Röter-Werden der Haut, ein Lauter- oder Leiser-Werden der Stimme, Anzeichen von Schwitzen oder Frieren, Tränen in den Augen, usw. Es gibt Situationen, für die ein Kranker keine Worte (mehr) hat, in denen er aber dennoch einen Zuhörer braucht. Dem Schweigen zuzuhören, fällt leichter, wenn man sich fragt: „Was sagen mir die Tränen des Kranken? Was vermittelt mir seine Körperhaltung? Was höre ich aus seiner Atmung?“
- Wertfreies Zuhören bedeutet, sich den Aussagen des Patienten zuzuwenden, ohne das Gesagte zu bewerten oder infrage zu stellen [28].
- Zuhören sollte von einem *„Ja“ zur Klage* getragen sein. Dem Patienten verbal zu bestätigen, dass Tränen und Klagen in Ordnung seien, reicht nicht aus. Die heilende Kraft des Zuhörens wird sich nur dann entfalten, wenn der Patient auch von den nonverbalen Botschaften seines Gegenübers her *spüren* kann, dass er weinen und klagen *darf*!
- Letztlich beinhaltet Zuhören, das Gehörte nicht einfach nur zu hören (wie man etwa die Worte eines Nachrichtensprechers hört), sondern sich auf das, was man gehört hat, auch einzulassen, es im eigenen Handeln zu berücksichtigen.

Die Auseinandersetzung mit spirituellen Belangen ist meist von mehr Frage- als Ausrufezeichen geprägt. In Anwesenheit eines Menschen, der wertfrei und wertschätzend zuhört, gelingt es manchen Patienten, ihre Gedanken zu ordnen, zu präzisieren und miteinander in Verbindung zu setzen. Der Zuhörer wird zum Resonanzboden: Bislang Unausgesprochenes gewinnt an Konturen, sodass sich Betroffene eigene Bedürfnisse und Überzeugungen bewusst machen, gegebenenfalls auch Antworten auf bestimmte Fragen entwickeln können. Über seine Funktion als Resonanzboden hinaus wird der Zuhörer zum *Zeugen* der Erfahrungen des Patienten und damit zum Zeugen von dessen Existenz. Eine Erfahrung, die gerade angesichts spiritueller Fragen und Nöte in hohem Maße entlastet.

### Standhalten

Die Konfrontation mit chronischem Schmerz löst bei vielen Menschen verstärkt oder zum ersten Mal in ihrem Leben Fragen nach dem Sinn ihres Lebens aus. Die Sinnfrage zeigt sich in vielen Formen, verbirgt sich mitunter in Ausrufen wie „Warum nur habe ich diesen Schmerz?“; „Ach, das bringt doch alles nichts mehr.“ Einige Patienten fragen auch explizit: „Was hat mein Leben denn noch für einen Sinn?“ Behandelnde müssen auf Fragen dieser Art keine Antworten haben, doch sollten sie ihnen *standhalten* können. Oftmals ist die Frage nach dem „Warum?“ oder „Wozu?“ nicht eigentlich eine *Frage*, sondern ein *Aufschrei* angesichts der Erfahrung von Krankheit und Schmerz. An den Grenzen eigener Erkenntnis und Einflussnahme können Behandelnde oft wenig mehr tun, als einfach „da“ zu sein, auf nivellierende Vertröstungen ebenso zu verzichten wie auf die Vorgabe positiver Affirmationen. Wenn ein Patient beispielsweise fragt „Warum tut Gott mir das an?“ oder „Wozu das alles?“, verlangt er damit nicht, dass man ihm Gott und sein Handeln erklärt oder seinem Leiden einen Sinn gibt. Was er braucht, ist ein Mensch, der diese Fragen und das darin verborgene Leid aushalten kann. Standhalten ist keine Passivität, vielmehr höchste Aktivität:

„Es kostet auch den Begleitenden Kraft, das Aushalten-Müssen des Patienten aushalten zu können und ruhig zu begleiten, anstatt in Angebote auszuweichen, die zum aktuellen Zeitpunkt unrealistisch sind“ [41].

Standhalten basiert auf dem Konzept der Präsenz. Dieses Konzept, das in der Literatur vieler Fachdisziplinen zu finden ist [10], wird seit ca. 1960 vermehrt auch in der Literatur zu Therapie, Pflege und Seelsorge behandelt. Präsenz beinhaltet eben das, was das Wort besagt: präsent, d. h. für den anderen *da* zu sein. Nicht nur mit seinem Körper, auch mit seinem Verstand und seinem Herzen. Den Autoren Timmermann und Bart zufolge sind für eine Haltung der Präsenz v. a. entscheidend [52]:

- Die Bereitschaft, sich wiederholt in die Lebenswelt des anderen zu versetzen und sich darauf einzulassen
- Dabei zu bleiben, „auch wenn das Leiden oder die Situation aussichtslos ist“ (ebd.)
- Aufrichtig interessiert und sensibel zu sein – „nicht nur für die Frage des anderen und die Frage hinter der Frage, sondern auch für die Sehnsucht und den Appell, der von dem anderen ausgeht“ (ebd)

Wie Untersuchungen zeigen, ist die Haltung der Präsenz nicht nur für den Aufbau einer vertrauensvollen Beziehung zum Patienten entscheidend, sie bereitet zudem den Boden für zukünftige Veränderungen [15].

Wird ein Mensch chronisch schmerzkrank, geraten mehrere Säulen seiner Identität ins Wanken: seine leibliche Integrität, seine Arbeits- und Leistungsfähigkeit, seine Werte, seine materiellen Sicherheiten sowie mitunter auch sein soziales Netz. Die nicht nur körperliche, sondern auch emotionale Anwesenheit einer Fachperson, ihre unerschütterliche Präsenz auch dann, wenn der Patient von seiner Verzweiflung spricht, kann auf diesen wie ein Garant dafür wirken, dass er nach wie vor ein Mensch ist, der Beachtung findet. Denn letztlich bedeutet Standhalten nichts anderes als dies: *den Patienten in seiner Existenz zu bestätigen*. Eben darin liegt Trost.

### Aktivieren von Werten

Die Frage eines Menschen nach dem Sinn ist eine zutiefst spirituelle Frage, gilt doch das Sinnfinden „als zentrales Merkmal von Spiritualität“ [23]. Die Suche nach dem Sinn eines Lebens mit chronischem Schmerz ist ein individueller Weg und somit immer auch eine Frage nach der Identität des Menschen: „Was für ein Mensch will ich sein – mit diesem Schmerz?“ Antworten auf diese Frage erwachsen aus den Werten des Kranken, aus seiner Verbundenheit mit dem, was sein Leben trägt, ihm Orientierung und Halt gibt. Zur Aktivierung von Werten dienen Fragen wie [14]:

- Welche Eigenschaften, Interessen und Anliegen machen Ihre Person aus?
- Worauf in Ihrem Leben sind Sie heute noch stolz?
- Was möchten Sie auf keinen Fall anders haben?
- Wer oder was hat Sie durch frühere Krisen getragen?
- Was soll Ihren Angehörigen und Freunden von Ihnen in Erinnerung bleiben?

Die Aktivierung von Werten fördert das Bewusstsein des Patienten für das, was den Kern seiner Person ausmacht, stärkt sein Vertrauen, dass sein Leben Sinn und Bedeutung behält – unabhängig davon, ob seine Funktionstüchtigkeit besser, schlechter oder gleich bleiben wird.

Vor allem zu Beginn einer Therapie fällt es vielen Schmerzpatienten jedoch schwer, sich auf ihre Werte einzulassen. Im Vordergrund steht das, was verloren ist. Oft überwiegen Gefühle der Hoffnungslosigkeit und Verzweiflung. Informationen über die schmerzverstärkende Wirkung negativer Gefühle verbunden mit einer Schulung in positiven Affirmationen reichen nicht aus, um existenzieller Verzweiflung entgegenzuwirken. Leid will zunächst gewürdigt, d. h. wahrgenommen und anerkannt werden. Leid, das gesehen wird, wird erträglicher. Zugleich wird die Voraussetzung dafür geschaffen, dass sich der Betroffene in einem zweiten Schritt auch wieder positiven Aspekten seiner Person und seines Lebens zuwenden kann.

„Wie die Würdigung von Leiderfahrungen und die Bewusstmachung von Werten ineinandergreifen können, soll im Folgenden beschrieben werden – anhand der Frage eines Patienten: ‚Was hat mein Leben denn jetzt noch für einen Sinn?‘ Auf diese Frage mit einer direkten Frage nach seinen Werten zu reagieren, könnte vom Betroffenen als Ausdruck unzureichender Würdigung seiner Leiden empfunden werden. Der erste Schritt besteht daher darin, aufzugreifen und anzusprechen, was aus der Frage des Kranken herauszuhören ist: Verzweiflung, Hoffnungslosigkeit, vielleicht auch Resignation.

Therapeut (Th): Aus Ihrer Frage höre ich große Trauer heraus, Hoffnungslosigkeit, auch Verzweiflung. So viel Leid, das Sie in den letzten Jahren erfahren haben! … Wäre es in Ordnung, einmal eine Bestandsaufnahme von all dem Dunklen in Ihrem Leben zu machen? Zu notieren, was Ihnen widerfahren ist? Hier ist ein Blatt Papier. Hier sind Farb- und Bleistifte. Erst einmal zeichne ich einen Kreis auf das Papier und unterteile den Kreis in zwei gleich große Bereiche … Nun möchte ich Sie bitten, sich ganz auf Ihre Verzweiflung zu konzentrieren, auf Ihre Trauer über das, was Sie verloren haben. Vergegenwärtigen Sie sich all das, was Sie als dunkel und belastend in Ihrem Leben erleben … Nun versuchen Sie, Ihren Gedanken und Gefühlen Ausdruck zu geben – mit einem kurzen Satz, einem Schlagwort oder mit einem Symbol, also mit Farben und Formen. Zeichnen, malen oder schreiben Sie in einen der beiden Bereiche des Kreises, was Ihnen spontan dazu einfällt.

(Kleine Pause, während der Patient schreibt oder malt.)

Th: Bitte, lenken Sie Ihre Aufmerksamkeit nun auf das, was Sie als hell und heil in sich erleben, auf das, was Ihnen Kraft gibt oder Ihnen bei früheren Krisen geholfen hat. Wählen Sie wiederum eine Farbe, eine Form oder ein Wort, das dazu passen könnte. Malen oder schreiben Sie Ihre Worte oder Symbole in den zweiten Bereich des Kreises.

Nachdem Therapeut und Patient das fertige Bild eine Weile betrachtet haben:

Th: Woran bleibt Ihr Blick haften, wenn Sie auf Ihr Bild schauen? (Nachdem der Patient auf ein bestimmtes Wort oder Symbol gezeigt hat.) Wenn dieses Symbol sprechen könnte, was würde es sagen?“

Die gleichzeitige Vergegenwärtigung dunkler wie heller Aspekte stärkt das Bewusstsein des Kranken für die *Ganzheitlichkeit* des Lebens – dafür, dass Freude und Leid einander keineswegs ausschließen, vielmehr *nebeneinander* bestehen. Die Befürchtung, man könne das negative Erleben eines Menschen womöglich vertiefen, indem man ihm Aufmerksamkeit schenkt, ist unbegründet. Wie Untersuchungen zum „*affect labeling*“ zeigen, reduziert das Ansprechen negativer Gefühle die Aktivität in den emotionalen Zentren des Gehirns (v. a. in der Amygdala und in anderen limbischen Regionen), während sich die Aktivität im Großhirn (v. a. im präfrontalen Kortex) erhöht [31]: „Wenn also ein Gefühl nicht nur ein Gefühl bleibt, sondern mit Sprache ‚zum Ausdruck‘ kommen darf, erleben wir Menschen eine Stresslinderung und die Zunahme ‚klarer Gedanken‘“ [34]. Auch neurowissenschaftliche Befunde kommen somit zu folgendem Schluss: Der Weg zu den Kraftquellen eines Menschen fängt oft bei seiner Verzweiflung an.

Die Aufforderung, einem der Worte oder Symbole eine Stimme zu geben, basiert auf der psychodramatischen Technik des *Rollenwechsels* [33]. Ein Rollenwechsel mit einem Symbol aus dem „dunklen“ Bereich erleichtert es, Gefühle der Angst und Verzweiflung zu differenzieren. Indem der Betroffene seiner Angst eine Stimme gibt, ist er ihr nicht mehr hilflos ausgeliefert, geht vielmehr aktiv damit um. Im Rollenwechsel mit einem Symbol aus dem „hellen“ Bereich wird vielen Betroffenen bewusst, was sie zuvor – überrollt von Verzweiflung und Angst – nicht haben wahrnehmen können: dass es in ihrem Leben durchaus auch positive Erfahrungen gibt, Quellen der Kraft und der Sinngebung. Einige Patienten gewinnen Kraft aus der Liebe zu ihrem Partner, manche aus der Vorstellung, ihren Kindern modellhaft vorzuleben, dass Krankheit und Schmerz nicht das Ende eines erfüllten Lebens bedeuten. Wieder andere sehen eine Art Aufgabe darin, trotz anhaltender Schmerzen freundlich zu bleiben, offen für das, was in ihrer Umgebung geschieht [8].

### Rückgriff auf menschheitsgeschichtliche Motive

In *Mythen, Sagen, Legenden und Märchen* aller Zeiten und aller Kulturen finden sich zahlreiche Beispiele für Grenzerfahrungen menschlichen Lebens – auch für die Verarbeitung körperlicher und seelischer Schmerzen, für die Suche nach Sinn und Identität [53]. Für Menschen, die schwer an der Sinnlosigkeit und Ungerechtigkeit ihrer Erkrankung leiden, bieten sich Hinweise auf die Hiobsgeschichte oder das Buch Kohelet an – unabhängig davon, ob Behandelnde und Behandelte die Bibel als Wort Gottes oder als Kulturgut verstehen. Kohelet beispielsweise ist ein Mann, der verzweifelt nach dem Sinn des Lebens sucht und nichts als Ungerechtigkeit und Sinnlosigkeit findet. Diese Erkenntnis endet jedoch nicht in Resignation, vielmehr in Akzeptanz und Gelassenheit: „Genieße dein Leben, bevor es zu Ende geht“ (Kohelet 12,6).

Menschen, die einen Bezug zu Opern haben, könnten an ein Zitat aus „Der Rosenkavalier“ von Richard Strauss erinnert werden:

„Das alles ist geheim, so viel geheim.

Und man ist dazu da, dass man’s ertragt.

Und in dem ‚Wie‘ da liegt der ganze Unterschied.“

Wieder andere Patienten finden ihr Erleben treffend zusammengefasst in einem Song von Janis Joplin: „Freedom is just another word for nothing left to lose“ oder einem Song von Udo Lindenberg: „Ich geh meinen Weg/Ob gerade ob schräg, das ist egal.“

Auch *Bildhauer und Maler* haben Krankheit und Schmerz wiederholt zu ihrem Thema gemacht (Vincent van Gogh, Käthe Kollwitz, Edvard Munch usw.). Frida Kahlo beispielsweise hat ihren Schmerz in all seinen Facetten sichtbar gemacht, auch im Bett liegend, auch unter stärksten Schmerzmitteln und Drogen. Am eindrücklichsten ist wohl ihr Bild „Die zerbrochene Säule“ (https://www.kahlo.org/de/die-gebrochene-saule/).

Die Heilkraft der *Musik* haben sich Menschen bereits in vorgeschichtlicher Zeit zunutze gemacht. Inzwischen ist ihre Wirkweise bei verschiedenen Patientengruppen untersucht und bestätigt worden [49]. In jüngster Zeit wächst auch die Literatur zum Thema *Musik und Spiritualität*, insbesondere im Rahmen palliativer Versorgung [55]. Untersuchungen zum Einsatz der Musiktherapie bei chronischem Schmerz zeigen „signifikante Verbesserungen der Schmerzen, der Schlafqualität, des allgemeinen Befindens und eine deutliche Reduktion von Angst und Depression“ [51]. Studien zum Einsatz der Musiktherapie bei *spirituellen* Themen im Rahmen multimodaler Schmerztherapie stehen noch aus.

Mit der Einbeziehung menschheitsgeschichtlicher Motive ist *nicht* gemeint, dass in der Schmerztherapie tätige Menschen über detaillierte Kenntnisse in Religion, Mythologie, Literatur, Malerei und Musik verfügen sollten. Worum es vielmehr geht: Motive zu verwenden, die das Erleben des Patienten in verdichteter Form zum Ausdruck bringen, ohne ihn zu belehren. Auf diese Weise wird ihm vermittelt, dass viele seiner Anliegen zu den großen Fragen der Menschheit gehören – in allen Kulturen, zu allen Zeiten. Diese Erkenntnis ändert nichts an seinem Schmerz, lindert jedoch seine Einsamkeit. Wir leiden nicht allein. Wir quälen uns nicht allein mit Fragen nach dem Sinn von Krankheit, Schmerzen und Tod. Und darin liegt Trost.

Wie der Rückgriff auf menschheitsgeschichtliche Motive konkret aussehen könnte, sei an einem Beispiel verdeutlicht:

„Herr F., 58 Jahre alt, macht sich Vorwürfe, dass er seine chronischen Schmerzen nicht klaglos akzeptieren kann:

Pt: Ich bin so wütend auf Gott, dass er mir diesen Schmerz geschickt hat! Ich kann gar nicht mehr beten. Das macht mir zu schaffen. Denn als guter Christ müsste ich mein Kreuz doch eigentlich auf mich nehmen, nicht wahr?

Th: Herr F., gerade muss ich an das denken, was Jesus am Kreuz gerufen hat, kurz vor seinem Tod. Ich weiß nicht, ob Sie sich vielleicht erinnern?

Pt: Ja, natürlich. Er rief ‚Mein Gott, warum hast du mich verlassen?‘ (hält inne, schaut die Therapeutin erstaunt an): Oh, das hatte ich vergessen. Jesus zweifelt ja auch an Gott. Er sagt gar nicht: ‚Alles gut.‘ Nein, er fühlt sich verlassen von Gott. So, wie *ich* mich jetzt verlassen fühle von Gott.

Th: Was bedeutet das für Sie, Herr F.?

Pt: Ich kann wohl nicht fester im Glauben stehen als Jesus. Wenn Jesus das sagen oder sogar schreien konnte, dann kann ich das vielleicht auch?

Th: Vielleicht?

Pt (lächelt): Nein, nicht vielleicht.

Th: Möchten Sie es jetzt einmal ganz laut sagen?

Pt (Tränen laufen über sein Gesicht): Mein Gott, warum hast du mich verlassen?!

Th: Wie fühlt sich das an?

Pt: Was ich gerade merke: Ich zweifle ja gar nicht an Gott. Also, wenn ich ihn anschreie, dann könnte ich das ja eigentlich nur, weil ich sicher bin, dass es ihn gibt.

Th: Und? Sind Sie sicher?

Pt (entschieden): Ja. Ich *bin* sicher. Ich fühle Gott wieder. Ich *verstehe* ihn nur nicht. Aber ich weiß, dass er *da* ist.“

Die Erinnerung daran, dass Jesus sein Leid keineswegs gelassen und abgeklärt auf sich genommen hat, sondern mit einem Schrei der Verzweiflung, wirkt auf Herrn F. in hohem Maße entlastend. Diese Entlastung hält an, wie im Laufe seiner weiteren Behandlung deutlich wird.

In der Begegnung mit schmerzkranken Menschen brauchen wir alles, was wir je gesehen, gelesen und gehört haben. Aus diesem Fundus kann ein jeder von uns das schöpfen, was zu dem jeweiligen Patienten passt – zu seiner Individualität und Besonderheit.

## Die erweiterte Rolle der Fachperson

Die Funktion von Fachpersonen beim Umgang mit der Spiritualität chronisch schmerzkranker Menschen ist eine andere als beim interprofessionellen schmerztherapeutischen Umgang mit ihrer Schmerzerkrankung im Rahmen der bisher angewendeten multimodalen Therapieansätze [4].

Im Hinblick auf die spirituellen Anliegen und Bedürfnisse eines Patienten hat der Behandelnde oft nichts anderes anzubieten als sich selbst – seine nicht nur körperliche, sondern auch seelisch-geistige Anwesenheit. Angesichts eines verzweifelt weinenden Menschen ist die Versuchung groß, ein Medikament zu verordnen, diese oder jene Bewältigungsstrategie vorzuschlagen [29]. Nur: Es gibt Situationen, da hilft weder das eine noch das andere. Was aber hilft: das Leid des Patienten wahrzunehmen, zum mitfühlenden Begleiter zu werden, zumindest für einen Moment. Die unerschütterliche Präsenz des Behandelnden auch dann, wenn ein Patient nach dem Sinn seines Lebens fragt, kann auf diesen wie ein Garant dafür wirken, dass es vielleicht doch einen Sinn gibt, auch wenn er selbst derzeit keinen Sinn sieht.

## Fazit für die Praxis

- Dreh- und Angelpunkt für den Umgang mit der Spiritualität eines schmerzkranken Menschen ist die Herstellung einer Beziehung, in der er sich akzeptiert und angenommen fühlt. Diese Beziehung ist Voraussetzung dafür, dass ein Gespräch über spirituelle Belange überhaupt stattfinden kann. Die Rolle des Behandelnden erweitert sich vom Experten zum Begleiter.
- Zentrale Einstellungen und Verhaltensweisen zur Herstellung einer Beziehung, in der sich ein Patient für spirituelle Themen öffnen kann, wurden beschrieben. Wichtig vor allem ist die Bereitschaft des Behandelnden, dem Leid des Betroffenen standzuhalten, eine Weile bei ihm zu bleiben – nicht nur mit seinem Körper, sondern auch mit seinen Gedanken.
- Künftige Studien sollten die Effizienz der hier diskutierten Interventionen überprüfen, um klarer herausarbeiten zu können, welche Aspekte für einen biopsychosozial-spirituellen Therapieansatz im Einzelnen bedeutsam sind.
